# Non‐Invasive Monitoring of Hazel Dormouse (*Muscardinus avellanarius*): First Insights on Genetic Data Quality From the First Pilot Study

**DOI:** 10.1002/ece3.72622

**Published:** 2025-12-08

**Authors:** Francesco Mezzetti, Martina Muraro, Maria Chiara Fabbri, Francesca Maura Cassola, Edoardo Velli, Cristiano Tabarroni, Jacopo Lorusso, Nadia Mucci, Roberto Cazzolla Gatti, Matilde Martini

**Affiliations:** ^1^ Unit for Conservation Genetics (BIO‐CGE), Department for the Monitoring and Protection of the Environment and for Biodiversity Conservation Italian Institute for Environmental Protection and Research (ISPRA) Bologna Italy; ^2^ Department of Biology, Life and Mind Building University of Oxford Oxford UK; ^3^ Department of Agriculture, Food, Environment, and Forestry (DAGRI) University of Florence Florence Italy; ^4^ School of Life Sciences, Faculty of Science and Engineering Anglia Ruskin University Cambridge UK; ^5^ Department of Biological, Geological and Environmental Sciences University of Bologna Bologna Italy

**Keywords:** hair samples quality, hair tubes, hazel dormouse, microsatellite loci, non‐invasive sampling, wildlife monitoring

## Abstract

Genetic non‐invasive sampling (gNIS) methods, which enable DNA extraction from naturally shed materials, offer a powerful, low‐impact tool for studying elusive species, minimising disturbance and ethical concerns. However, such approaches require careful assessment of sample quality and data accuracy to ensure reliable genetic results. The hazel dormouse ( *Muscardinus avellanarius* ) is an arboreal rodent that plays a crucial role in ecosystems. Despite conservation concerns arising from its low population densities, which are closely linked to habitat quality, it remains poorly understood due to monitoring challenges. This pilot study, conducted in the high Agri Valley (Basilicata, Italy), evaluates a non‐invasive genetic sampling protocol for the hazel dormouse using hair‐tubes. We investigated the genotyping efficiency, the reliability and informativeness of selected microsatellite markers and the impact of hair sample quantity and trap placement duration on genotyping success and error rates. We used 126 hair tube traps at 14 sampling sites, obtaining reliable genotypes for 14 individuals out of 32 hair samples collected, with a genotyping efficiency significantly affected by both the number of hairs collected and the collecting time interval. The microsatellite marker panel showed a high number of alleles and a low probability of identity, proving to be informative for population monitoring. The results underscore the potential of this non‐invasive approach for monitoring elusive and conservation‐priority species while highlighting the need to minimise field collection time and optimise trap design to improve DNA quality and genotyping reliability.

## Graphical abstract

We evaluated a non‐invasive genetic sampling protocol for the hazel dormouse ( *Muscardinus avellanarius* ) using hair tubes deployed at 14 sites in the high Agri Valley to assess DNA degradation and individual genotyping efficiency. A seven‐locus microsatellite panel was highly informative and provided adequate power for individual discrimination, but genotyping success declined with low hair quantity and prolonged trap exposure. These results offer a methodological framework for monitoring *M. avellanarius* and practical guidance for designing genetic‐sampling protocols for elusive small mammals.

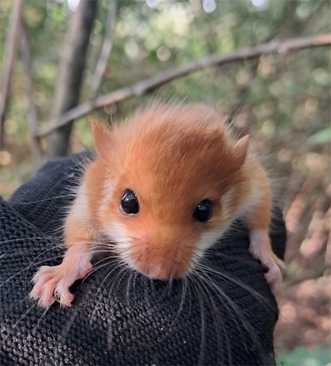

## 1 Introduction

Genetic non‐invasive sampling methods (gNIS) have become essential tools in ecology and evolutionary studies (Ferreira et al. 2018), offering a broad range of applications, including population size and demography rate estimation, assessments of gene flow and connectivity, disease surveillance, bioaccumulation and even behaviour analysis (Squadrone et al. 2022; Waits 2005). gNIS relies on extracting DNA from naturally shed materials such as scat, feathers, urine or hair (Taberlet and Luikart 1999) and has been effectively applied across a wide range of taxa, including insects, amphibians, reptiles, birds and mammals (Waits 2005). Despite its advantages, gNIS also presents challenges. DNA extracted from non‐invasive samples is often degraded due to environmental factors such as ultraviolet radiation, moisture and enzymatic activity from moulds and bacteria (Santini et al. 2007). This degradation can reduce genotyping success and accuracy, increasing genotyping errors such as allelic dropout (ADO) (Valière et al. 2007). Consequently, studies on non‐invasive sampling methods should include assessments to evaluate sample quality and the accuracy of the results.

**FIGURE 1:**
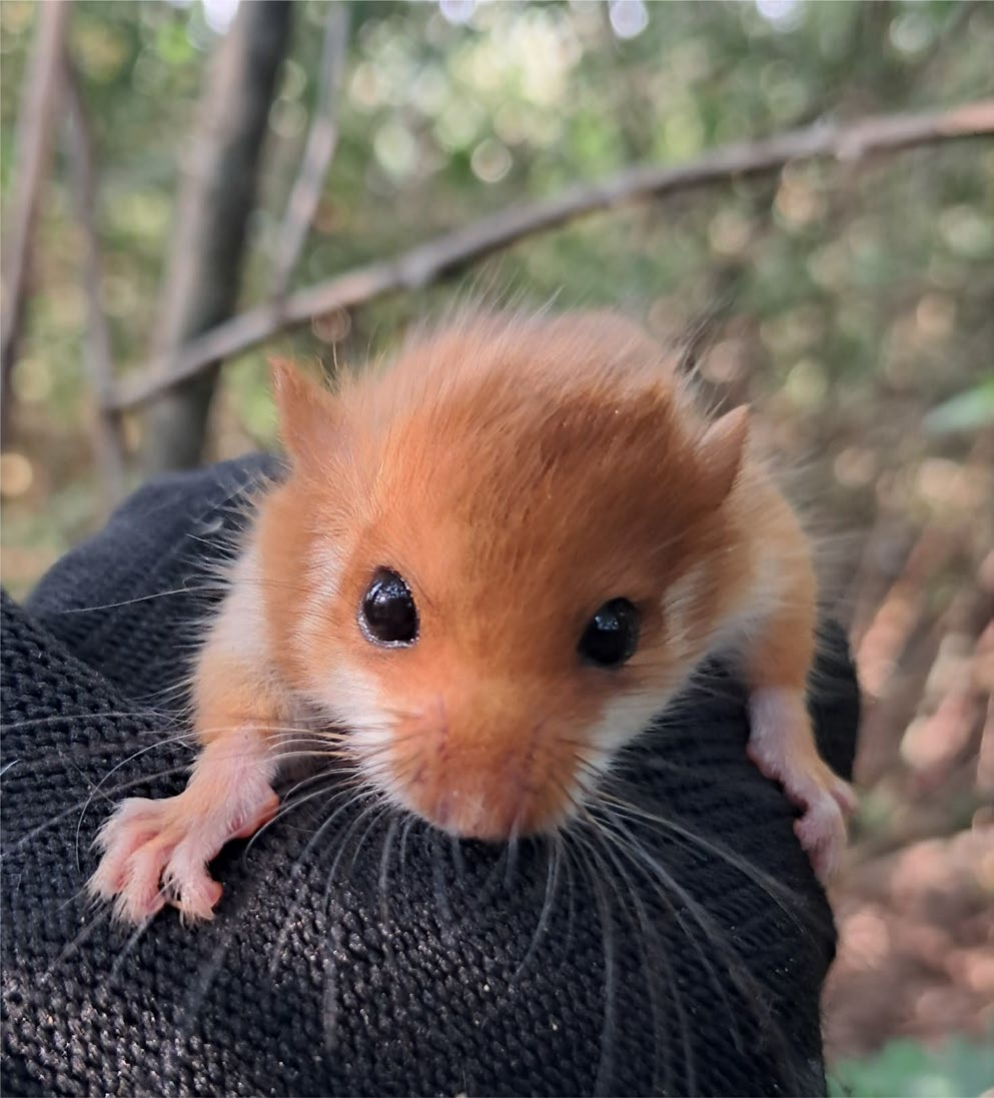
Adult hazel dormouse ( *Muscardinus avellanarius* ) in its natural habitat. This small arboreal rodent, typical of European deciduous forests, is included in Annex IV of the EU Habitats Directive.

Among mammals, small rodents play crucial roles in ecosystems (Lacher et al. 2016), but despite their ecological and conservation importance, our knowledge remains limited (Verde Arregoitia 2016) since monitoring their population dynamics and genetic patterns is not trivial, as they are hard to detect and sample (Ferreira et al. 2018). Traditional small mammals sampling methods based on live trapping are labour‐intensive, resource‐demanding and ethically concerning due to the significant stress and potential injury inflicted on sensitive or threatened species (Powell and Proulx 2003). gNIS applied to small mammals' hairs collected using hair tubes provides an effective and cost‐efficient alternative for sampling rare and elusive species while minimising disturbance and ethical concerns compared to invasive methods (Ferreira et al. 2018; Valière et al. 2007). However, this method presents specific biological and technical challenges. The typically fine, short nature of hair, yielding low amounts of nuclear DNA (Reiners et al. 2011), together with DNA degradation resulting from environmental exposure and contamination from multiple hazel dormice individuals, complicates genotyping (Piggott and Taylor 2003). These factors make small mammals difficult subjects for gNIS and underscore the need for adapted strategies to ensure data quality and reliability. The hazel dormouse ( *Muscardinus avellanarius* ) (Figure 1), an arboreal rodent found primarily in deciduous and mixed woodlands but also occurring in hedgerows, is a species of European conservation concern due to its low population densities that are closely linked to habitat quality (Bani et al. 2017). Given these characteristics, applying genetic techniques to non‐invasively collected hair samples could provide valuable insights into population size, genetic connectivity and diversity, ultimately supporting conservation efforts for this species.

The population genetics of the hazel dormouse has been investigated in previous studies using both microsatellite loci and mitochondrial DNA to describe the genetic structure of populations and the main factors limiting gene flow between areas (Bani et al. 2017; Friebe et al. 2018; Mouton et al. 2017). However, these studies relied on DNA extracted from invasive sources, including captured animals, museum specimens or dead animals, and none of them have analysed DNA extracted from biological traces recovered in the field. In contrast, non‐invasive methods have been successfully applied in other studies (Priestley et al. 2021) but have not been applied to population genetics for this species. Consequently, no study has yet examined the combined use of hair tubes in the context of population genetics while accounting for sample quality and DNA degradation. To address this gap, pilot studies are essential to assess the feasibility of this approach under field conditions.

In this study, we conducted a pilot assessment of non‐invasive genetic sampling of *M. avellanarius* using hair tubes. We aimed to, (i) assess genotyping efficiency by determining the proportion of successfully genotyped samples and identifying the minimum number of loci required for individual identification, (ii) determine the reliability and informativeness of selected microsatellite markers by analysing polymorphic information content (PIC), and amplification success across loci, and (iii) explore limitations potentially due to DNA degradation by assessing the effects of hair quantity and collection time intervals on genotyping success and error rates associated with allelic dropout (ADO) and false alleles (FA). Additionally, we aimed to establish best practices for sample collection, storage and marker selection to optimise future non‐invasive genetic studies.

## 2 Materials and Methods

### 2.1 Hair Tube Design

**FIGURE 2:**
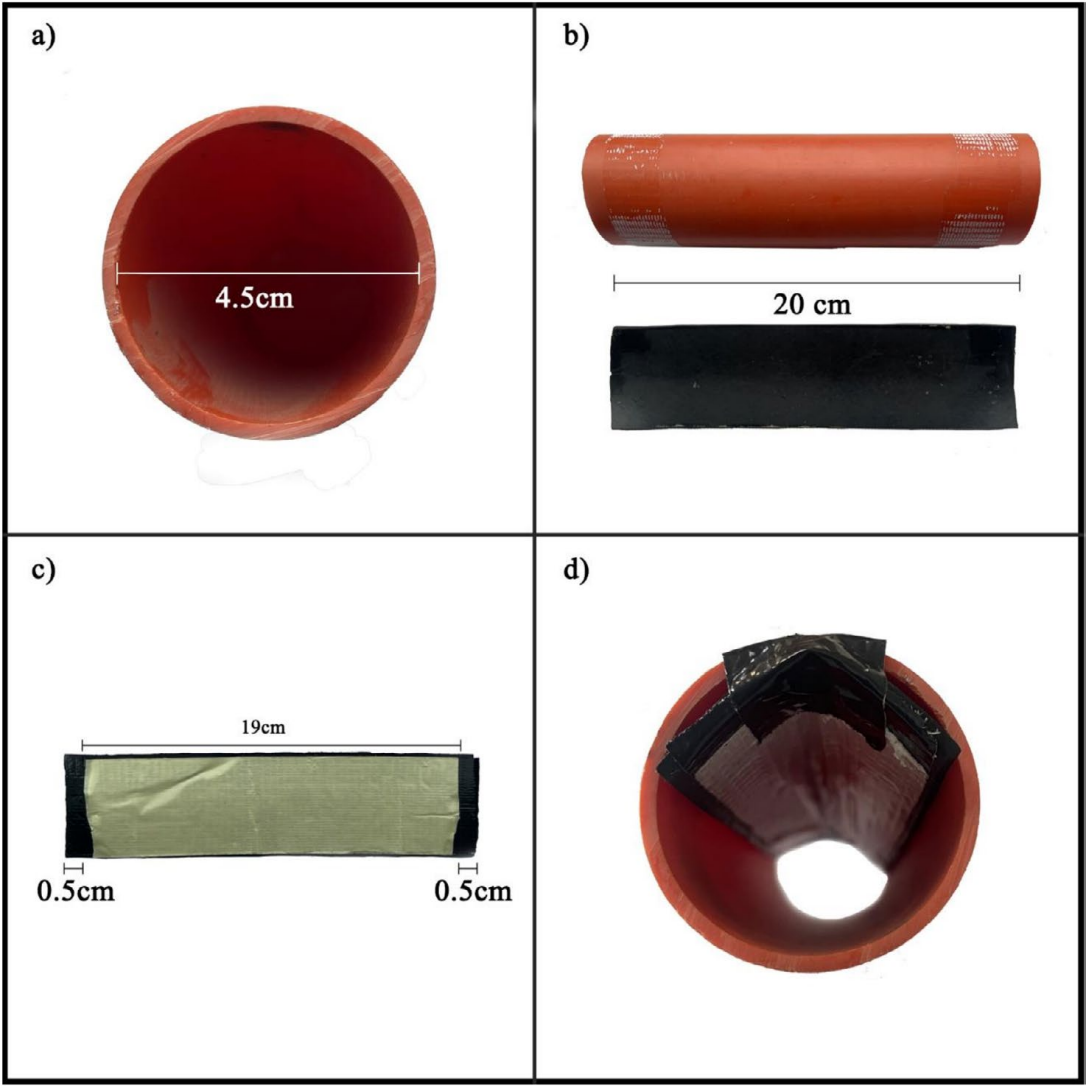
Overview of the materials and assembly process for the non‐invasive sampling method. The hair tube consists of: (a) a 20 cm‐long PVC tube (⌀ 4.5 cm); (b) a rubber base (5 × 20 cm); (c) a 20 cm‐long piece of duct tape which is applied to the rubber base, followed by a 19 cm‐long strip of double‐sided tape placed on top, leaving a 0.5 cm margin on both ends to facilitate removal after sampling. (d) The assembled base is then positioned at the top end of the PVC tube and secured using two additional strips of duct tape.

To collect hair samples from the hazel dormouse, we used an indirect, non‐invasive sampling method known as hair‐tubing, which is effective for arboreal mammals (McCleery et al. 2022). To optimise the collection process, we designed an easy‐to‐assemble hair tube structure that allows for effective hair sample collection and retrieval. We developed hair tubes with a diameter of 4.5 cm (Figure 2a), consisting of two main components: a 20 cm‐long PVC tube and a rubber base measuring 5 × 20 cm (Figure 2b). A 20 cm‐long piece of duct tape is attached to the rubber base, and a 19 cm‐long strip of double‐sided tape is placed on top, leaving a 0.5 cm free gap on both the right and left sides (Figure 2c). This gap facilitates the removal of the duct tape. The rubber base is then placed at the top of the PVC tube and secured with two strips of duct tape (Figure 2d). This hair tube setup allows for height adjustment of the rubber base, making the device adaptable to individuals of different body sizes. Hazel dormouse hairs can be easily collected by removing the protective layer of the double‐sided tape. When the animal enters the hair tube, attracted by the bait, its dorsal hairs stick to the adhesive strip.

### 2.2 Field Sampling Design

We collected hair samples of hazel dormouse between May and August 2023 at 14 sampling sites, covering a total area of 249 km^2^ in the high Agri Valley, in the Basilicata Region (Southern Italy; coordinates: 40.3336, 15.9062). The sampling sites were selected within deciduous woodland patches, primarily dominated by *Quercus cerris* , with secondary species including *Acer campestre* , *Fraxinus ornus* and *Quercus pubescens*, from 580 to 950 m above sea level. At each sampling site, a 3 × 3 grid of hair tubes was set up, with a total of nine tubes. Each line of the grid was composed of three tubes spaced 20 m apart along each line, maintaining a distance of 10 m between lines. Each hair tube was baited with hazelnut chocolate and hazelnuts or walnuts. Hair tubes were initially placed in May, and the first hair collection was conducted after an average of 70.8 ± 0.9 days (range 66–76). During this first visit, the bait was replaced, and new double‐sided tape was applied. A second sampling collection took place after an average of 41.2 ± 0.3 days (range 39–44), to evaluate a possible correlation between DNA degradation and the duration of hair exposure in the trap.

### 2.3 Hair Sample Collection and Preservation

During each check, the tape containing the hair samples was visually examined for preliminary species identification. The dorsal hazel dormouse hairs are typically very fine and vary in colour from yellowish‐brown to orange, measuring 9–10 mm in length, which makes them easily distinguishable from those of other small mammals (Paolucci and Bon 2022). However, they may sometimes be confused with *Mustela nivalis* hairs due to their similar colouration, but the greater length (≈15 mm) and thickness of the latter significantly reduce the possibility of misidentification.

Once the dormouse hairs were identified, they were collected by carefully removing the duct tape along with the double‐sided tape from the bottom of the tube. Each strip with hair samples was stored and transported in a transparent plastic bag and processed immediately after collection. Each bag was labelled with the site ID, and sample collection date.

To facilitate hair removal from the adhesive double‐sided tape without damaging its structure, a 1:1 solution of water and ethanol was applied dropwise using a pipette. The hairs were then carefully collected using fine tweezers and a needle and subsequently preserved in an Eppendorf 2.0 mL tube containing 1 mL of ethanol. Instruments were decontaminated after each operation to avoid cross‐contamination, and samples were shipped to the Institute for Environmental Protection and Research (ISPRA), where they were stored at −20°C until DNA extraction.

### 2.4 DNA Extraction and Genotyping

DNA was extracted using the Qiagen DNeasy Blood & Tissue Kit on a QIAcube HT robotic station (Qiagen Inc., Hilden, Germany). Each air‐dried hair sample was put in a 1.5 mL safe‐lock tube containing 180 μL of ATL lysis buffer and 20 μL of proteinase K. Enzymatic digestion to solubilise cell membranes and protein structures was carried out at 56°C overnight. All subsequent extraction steps were carried out on a QIAcube HT, following the manufacturer's instructions.

DNA samples underwent an initial screening following the multi‐tube approach, consisting of four independent amplifications per sample and per locus to minimise genotyping errors due to ADO and FA (Taberlet et al. 1996). Samples were amplified by Polymerase Chain Reaction (PCR) at three loci (Mav1, Mav3 and Mav4; Table 1), selected for their high allele number and short amplicon lengths (Bani et al. 2017). Samples with more than 50% successful PCRs (Caniglia et al. 2012) were further amplified, following the same multi‐tube procedure, at five additional loci (Mav2, Mav5, Mav6, Mav7 and Mav8) (Bani et al. 2017; Table 1). PCR amplifications were performed using the Multiplex PCR Kit (Qiagen Inc., Hilden, Germany) in a 10 μL reaction volume (5 μL of MasterMix, 1 μL of Q‐Sol, 1.8 μL of RNase‐free water, 2 μL of DNA and 0.20 μL of each 10 μM primer). Amplification conditions included 45 cycles with a final extension at 72°C for 10 min and different annealing temperatures as specified in Table 1. Negative and positive control samples were used to detect any potential DNA contamination event, and each step of the analysis was conducted in dedicated rooms under UV‐sterilised hoods. Where possible, liquid handling steps were performed by a Tecan EVO 100 Workstation (Tecan‐UK, Reading, UK).

**TABLE 1:** Loci used in this study (Bani et al. 2017).

Locus	Primer sequence (5′ → 3′)	Ta (°C)
*Mav1* ^a^	F: ATAGCCCAGAGGTAGAAA R: TAGCATCCCGTTCTCAAACC	57
*Mav2*	F: GAAGGGCTGGGTATATAT R: GCCTCCACCCAGAGACCG	60
*Mav3* ^a^	F: CACATGTGTTGACTGATT R: TCATAGGTTTGCGTCCAGCC	57
*Mav4* ^a^	F: GTAGAGCTGAGGGTATAACTTGG R: GCTGGTATTGCATGAGACG	57
*Mav5*	F: CCATTGGTCCAAGCCACA R: GAAAGAACCTCAATTAAAGC	60
*Mav6*	F: TCTTGCCTCGAAATGACT R: AGGTGTAAGGGTATAGCTTGG	60
*Mav7*	F: AAGTTGCTTGGTCTCTTTGG R: GCTAAGACTCAAACCCAAGGC	60
*Mav8*	F: GTGTAGCTTGAAGGTAGA R: AAACTTGGTACTAGTGCAGAC	60

Abbreviation: Ta, annealing temperature.

^a^ Loci selected for screening.

Amplicons were separated using an ABI Prism 3500xl Genetic Analyser and allele sizes were scored using GENEMAPPER v.6 (Thermo Fisher Scientific). Consensus genotypes were reconstructed using GIMLET 1.3.3 (Valière 2002). Given the high risk of contamination in hair trap sampling, each allele was recorded only if observed at least twice (Taberlet et al. 1996), and loci showing more than two alleles, interpreted as potential mixed samples, were discarded.

The minimum number of loci required for individual identification was assessed using the probability of identity among sibling index, P_(ID)sibs_, calculated in GeneAlEx 6.5 (Peakall and Smouse 2012). The reference threshold was chosen following Waits et al. (2001). The polymorphic information content of the loci (PIC) was assessed following Nagy et al. (2012).

### 2.5 Genotyping Success and Error Rate

To correlate hair samples quality with genotyping success and error rates (ADO and FA), samples were grouped into four categories based on the number of hairs: less than 5 hairs (A), 5–9 hairs (B), 10–15 hairs (C), and more than 15 hairs (D, Table 2). To evaluate the effect of the duration of sample permanence in the trap on genotyping success, samples were categorised based on the number of days between trap deployment and hair collection. A cut‐off of 45 days was used, corresponding to the maximum exposure time observed during the second sampling session (range: 39–44 days), which was designed to assess short‐term DNA degradation. Samples collected within this interval were assigned to the Short Time group (ST, ≤ 45 days), while those collected after longer exposure periods, up to a maximum of 76 days (range: 66–76 days; first sampling session), were assigned to the Long Time group (LT, > 45 days; Table 2). The selected exposure times were chosen to evaluate sample quality within a commonly applied timeframe for the hazel dormouse (Capizzi et al. 2002; Martini et al. 2024), which may vary depending on research objectives (Sozio et al. 2016), aiming to achieve a balance between field effort and data quality when using gNIS methods. The incidence of single and combined factors (samples' hair quantity and permanence time inside the tube traps) was investigated using a Fisher's exact test to identify a significant correlation with genotyping success. Differences in mean error rates were tested using non‐parametric Kruskal–Wallis and Mann–Whitney *U* tests, respectively, to investigate hair quantity and collection time.

**TABLE 2:** Percentage of positive PCRs, number of alleles (*N*_a_) and polymorphic information content (PIC) by locus. The percentage of positive PCRs was calculated for all samples that passed the screening.

Loci	% positive PCRs	*N*_a_	PIC
Mav1	93%	11	0.86
Mav2	89%	9	0.82
Mav3	78%	8	0.80
Mav4	96%	9	0.81
Mav5	83%	9	0.84
Mav7	93%	7	0.75
Mav8	86%	6	0.68

## 3 Results

### 3.1 DNA Genotyping

Of the 126 hair tube traps deployed during the study, 32 contained hairs, resulting in a 25.4% trapping success rate. The total number of collected hairs with bulbs, which are the source of DNA, ranged from 1 to 45, with an average of 11.9 ± 1.8 (*n* = 32) per trap. Overall, a total of 381 hairs were collected during the study.

Eighteen out of 32 dormouse DNA samples (59%) passed the initial screening, and 15 were successfully genotyped at the entire seven‐loci panel (47%). However, one sample exhibited more than two alleles at five loci, possibly resulting in a mixed sample belonging to more than one individual and was discarded from downstream analyses. The remaining 14 samples (44%) were considered reliable genotypes, each corresponding to a unique individual.

Excluding Mav6, which was monomorphic, all microsatellite loci were polymorphic, with allele numbers ranging from 6 (Mav8) to 11 (Mav1) (Table 2). Mav1 exhibited the highest polymorphic information content (PIC = 0.86) followed by Mav5, Mav2, Mav4, Mav3, Mav7 and Mav8 (0.68) (Table 2). P_(ID)_ and P_(ID)sib_ values were respectively equal to 1.2 × 10^−9^ and 7.0 × 10^−4^. When examining an increasing locus combination, a value below the threshold identified by Waits et al. (2001; 4.2 × 10^−3^) was reached considering only the first five loci.

### 3.2 Genotyping Success and Error Rate

The percentage of successful PCR amplifications varied among loci, ranging from 96% for Mav4 to 78% for Mav3. The mean and standard error of ADO and FA rates across all loci were 0.42 (SE = 0.04) and 0.06 (SE = 0.01), respectively.

Effects influencing the genotyping success were investigated (hair quantity and collection time, Table 3), and a statistically significant impact was observed for both variables when considered individually (*p* < 0.01 for the number of hairs and *p* < 0.05 for trap duration). However, when these two variables were combined, no significant effect on genotyping success was detected, likely due to an unbalanced number of groups within the two clusters. Error rates were only marginally affected by these variables. ADO remained high across all groups (ranging from 0.21 to 0.50; mean = 0.36, SE = 0.06), while FA ranged from 0.00 to 0.11 (mean = 0.05, SE = 0.02). Neither hair quantity nor time of permanence in the hair trap tubes influenced the number of FA detected. However, a weak but statistically significant relationship (*p* = 0.045) was observed between trap duration and ADO rate, as determined by the Mann–Whitney *U* tests.

**TABLE 3:** Group name, attribute, number of samples analysed per group, percentage of samples passing the screening, number of successfully genotyped samples, and mean dropout and false allele rates among samples that passed the screening. Total samples: 32.

Group name	Attribute	Number of samples	Sample passing the screening (%)	Genotyping success (%)	Mean dropout rate	Mean false allele rate
A	< 5 hairs	8	25%	13%	0.43	0.00
B	5–9 hairs	7	43%	29%	0.21	0.11
C	10–15 hairs	8	63%	50%	0.50	0.06
D	> 15 hairs	9	89%	89%	0.32	0.03
LT	> 45 days of placement	14	36%	21%	0.55	0.05
ST	< 45 days of placement	18	72%	67%	0.29	0.05

## 4 Discussion

In this pilot study, we successfully tested the application of a non‐invasive genetic sampling protocol based on hair collection in hazel dormouse ( *M. avellanarius* ) populations.

Considering that a minimum of three to ten hair bulbs is required to minimise genotyping errors (Gagneux et al. 1997), the material obtained from our hair sampling tubes was sufficient, albeit limited. This underscores potential challenges associated with the current trap design, which should be addressed in future investigations.

Out of 32 hair samples collected, we reliably genotyped 14 individuals across seven microsatellite loci. Despite the preliminary nature of this study and the small sample size, the value of genotyping efficiency was comparable to those recorded for other species in gNIS projects (e.g., Caniglia et al. 2012). Furthermore, the genetic variability observed in this study was consistent with values described in Bani et al. (2017), despite variations in sampling methods (invasive vs. non‐invasive) and the number of samples analysed, suggesting that the non‐invasive protocol can efficiently describe the genetic pattern in hazel dormouse populations. The microsatellite markers employed showed different values of positive PCR amplification. Mav1, Mav4 and Mav7, which exhibited the highest amplification rates, appear particularly suitable as screening markers to enhance the initial selection strategy, reducing the number of unreliable genotypes discarded later in the process. While the marker panel is fully informative, genotyping errors, especially high ADO across all loci, were reported. Although the multitube approach can counteract the impact of genotyping errors on the consensus genotype reliability, testing additional markers (e.g., those proposed by Mills et al. 2013) could further enhance the efficiency of individual identification, reduce the impact of genotyping errors, and improve the genetic variability investigation.

The genotyping success achieved (47%) was influenced by both the amount of hair collected and the time elapsed between trap placement and sample collection. Increasing sample quantity and reducing retrieval time are, therefore, crucial for improving genotyping yields and reliability. In the case of mixed samples, low hair quantities per sample can compromise the process by providing insufficient material for repeated DNA extractions. We also investigated the correlation between the number of hairs collected and the duration of trap placement with genotyping error rates. Although hair tends to preserve high‐quality DNA better than other biological matrices, it often contains low quantities of nuclear DNA and is prone to degradation when exposed in the field for extended periods. Accordingly, we observed a statistically significant increase in ADO rates over time and a consequent decline in sample quality though a larger and more balanced sample size could provide more reliable insights into the influence of hair quantity and deployment time on genotyping error rates. Additionally, our genotyping error rates were higher than those reported in studies on species commonly investigated using gNIS (e.g., wolves or bears; Gervasi et al. 2024), for which well‐established protocols and marker panels are already available. In contrast, no standardised protocols currently exist for our target species, and factors that may contribute to an increased rate of ADO, such as primer binding site polymorphisms, PCR amplification conditions and preferential amplification, have not been fully investigated. However, it is more likely that shorter intervals between sample collection in those studies contributed to improved genotyping success, underscoring once again the importance of minimising inspection time to ensure consistent and reliable results.

This study shows that hair sampling tubes represent a viable non‐invasive technique for genetic analyses of hazel dormice and potentially other small mammals. To further enhance efficiency and improve the reliability of genotyping, we suggest increasing sample quantity through optimised hair trap design, minimising sampling time by refining field sample handling and further improving both the screening strategy and marker panel.

Overall, these results contribute to the development of a robust protocol for non‐invasive genetic sampling in *M. avellanarius* and offer broader guidance for applying gNIS methods in ecological studies of cryptic or low‐density micromammal species.

## Author Contributions

**Francesco Mezzetti:** conceptualization (equal), formal analysis (equal), investigation (equal), writing – original draft (lead). **Martina Muraro:** investigation (supporting), writing – original draft (supporting), writing – review and editing (equal). **Maria Chiara Fabbri:** writing – review and editing (equal). **Francesca Maura Cassola:** investigation (supporting), writing – review and editing (equal). **Edoardo Velli:** formal analysis (equal), writing – review and editing (equal). **Cristiano Tabarroni:** formal analysis (equal), writing – review and editing (equal). **Jacopo Lorusso:** investigation (equal), writing – review and editing (equal). **Nadia Mucci:** conceptualization (equal), supervision (equal), writing – review and editing (equal). **Roberto Cazzolla Gatti:** writing – review and editing (equal). **Matilde Martini:** conceptualization (lead), investigation (equal), methodology (equal), resources (lead), supervision (equal), writing – original draft (supporting), writing – review and editing (equal).

## Conflicts of Interest

The authors declare no conflicts of interest.

## Data Availability

Individual genotype and sampling data are openly available in Dryad at https://doi.org/10.5061/dryad.rbnzs7hrf.
